# Genomic evidence of spatially structured gene flow and divergent insecticide resistance backgrounds of the malaria vector *Anopheles funestus* in Tanzania

**DOI:** 10.1093/genetics/iyaf117

**Published:** 2025-07-07

**Authors:** Joel O Odero, Ismail H Nambunga, Hamis Bwanary, Gustav Mkandawile, John M Paliga, Salum A Mapua, Sophia H Mwinyi, Halfan S Ngowo, Nicodem J Govella, Emmanuel W Kaindoa, Frédéric Tripet, Anastasia Hernandez-Koutoucheva, Heather M Ferguson, Chris S Clarkson, Alistair Miles, David Weetman, Francesco Baldini, Fredros O Okumu, Tristan P W Dennis

**Affiliations:** Environmental Health and Ecological Sciences Department, Ifakara Health Institute, P. O. Box 53, Ifakara 67501, Tanzania; School of Biodiversity, One Health, and Veterinary Medicine, University of Glasgow, Glasgow G12 8QQ, UK; Environmental Health and Ecological Sciences Department, Ifakara Health Institute, P. O. Box 53, Ifakara 67501, Tanzania; Environmental Health and Ecological Sciences Department, Ifakara Health Institute, P. O. Box 53, Ifakara 67501, Tanzania; Environmental Health and Ecological Sciences Department, Ifakara Health Institute, P. O. Box 53, Ifakara 67501, Tanzania; Environmental Health and Ecological Sciences Department, Ifakara Health Institute, P. O. Box 53, Ifakara 67501, Tanzania; Environmental Health and Ecological Sciences Department, Ifakara Health Institute, P. O. Box 53, Ifakara 67501, Tanzania; Centre for Applied Entomology and Parasitology, School of Life Sciences, Keele University, Huxley Building, Keele, Staffordshire ST5 5BG, UK; Environmental Health and Ecological Sciences Department, Ifakara Health Institute, P. O. Box 53, Ifakara 67501, Tanzania; School of Biodiversity, One Health, and Veterinary Medicine, University of Glasgow, Glasgow G12 8QQ, UK; Environmental Health and Ecological Sciences Department, Ifakara Health Institute, P. O. Box 53, Ifakara 67501, Tanzania; Environmental Health and Ecological Sciences Department, Ifakara Health Institute, P. O. Box 53, Ifakara 67501, Tanzania; Environmental Health and Ecological Sciences Department, Ifakara Health Institute, P. O. Box 53, Ifakara 67501, Tanzania; Centre for Applied Entomology and Parasitology, School of Life Sciences, Keele University, Huxley Building, Keele, Staffordshire ST5 5BG, UK; Department of Epidemiology and Public Health, Vector Biology Unit, Swiss Tropical and Public Health Institute, Kreuzstrasse 2 Allschwil 4123, Switzerland; Department of Public Health, University of Basel, Petersplatz 1 Basel 4001, Switzerland; Genomic Surveillance Unit, Wellcome Sanger Institute, Wellcome Genome Campus, Hinxton CB10 1SA, UK; School of Biodiversity, One Health, and Veterinary Medicine, University of Glasgow, Glasgow G12 8QQ, UK; Genomic Surveillance Unit, Wellcome Sanger Institute, Wellcome Genome Campus, Hinxton CB10 1SA, UK; Genomic Surveillance Unit, Wellcome Sanger Institute, Wellcome Genome Campus, Hinxton CB10 1SA, UK; Department of Vector Biology, Liverpool School of Tropical Medicine, Liverpool L3 5QA, UK; Environmental Health and Ecological Sciences Department, Ifakara Health Institute, P. O. Box 53, Ifakara 67501, Tanzania; School of Biodiversity, One Health, and Veterinary Medicine, University of Glasgow, Glasgow G12 8QQ, UK; Environmental Health and Ecological Sciences Department, Ifakara Health Institute, P. O. Box 53, Ifakara 67501, Tanzania; School of Biodiversity, One Health, and Veterinary Medicine, University of Glasgow, Glasgow G12 8QQ, UK; Department of Vector Biology, Liverpool School of Tropical Medicine, Liverpool L3 5QA, UK

**Keywords:** population genetics, insecticide resistance, *Anopheles funestsus*, malaria, Cyp6p gene cluster, Cyp9k1, vector control, gene flow

## Abstract

Population genetic analysis of mosquitoes is important for understanding the distribution of insecticide resistance alleles, devising sustainable control approaches, and understanding how vector populations are structured in space. *Anopheles funestus* is the dominant malaria vector in most parts of East and Southern Africa. To better understand its population genetic structure in Tanzania, we sequenced the genomes of 334 individual *An. funestus* mosquitoes from 11 regions across the country. Signs of reduced migration between western and eastern cohorts across the semi-arid central region containing the Rift Valley suggest a partial barrier to gene flow between these populations. This was evidenced by population structure between the eastern and western cohorts, as well as asynchronous selective sweeps and copy number variant profiles at the *Cyp9k1* gene and *Cyp6p* gene cluster. Eastern cohorts, despite having less diversity and greater inbreeding, also share genetic histories characterized by low genome-wide *Fst* values with those in the west. This suggests that the barrier to gene flow is porous and likely represents continuous spatial structure rather than a complete barrier to migration. The observed population disconnectedness should be considered for insecticide deployment, resistance management, and the rollout of novel genetic-based vector control approaches. These findings provide the most detailed study of Tanzanian *An. funestus* population structure and resistance genetics to date. Future research should examine the epidemiological relevance of this partial discontinuity in gene flow and whether these populations have different malaria transmission abilities.

## Teaser

Odero et al. aimed to understand genetic diversity in *Anopheles funestus* mosquitoes across Tanzania to inform malaria control. The authors sequenced 334 mosquitoes from 11 regions, revealing population divisions into Eastern and Western mosquito groups that showed distinct genetic profiles at resistance genes, indicating limited migration between regions despite some connectivity. This informs targeted insecticide use and genetic-based control strategies. It provides crucial insights highlighting the need for further research on how these genetic differences affect malaria transmission dynamics.

## Introduction

*Anopheles funestus* is a dominant malaria vector in east and Southern Africa (Msugupakulya *et al*. 2023). Spanning a wide ecological range of tropical and sub-tropical Africa, it mediates most malaria transmission in Tanzania (Mapua *et al*. 2022; Matowo *et al*. 2023; Mwalimu *et al*. 2024), even in sites where it is outnumbered by other vector species like *Anopheles arabiensis* (Kaindoa *et al*. 2017; Mapua *et al*. 2022). Despite its epidemiological significance, the species has been less studied than other major African vectors, in part due to challenges in laboratory colonization, unresolved taxonomic complexities and challenges in field observations of the species in its natural habitats (Ngowo *et al*. 2021; Kahamba *et al*. 2022). The control of *An. funestus* is further complicated by widespread insecticide resistance (Hancock *et al*. 2020), following sustained use of insecticides in vector control [using insecticide-treated bed nets (ITNs) and indoor residual spraying (IRS)], and agricultural pest control (Churcher *et al*. 2016; Hemingway *et al*. 2016).

Previous studies have shown that ecological factors significantly influence population-level genetic structure in *Anopheles* species (Lehmann *et al*. 2003; Pinto *et al*. 2013). *Anopheles funestus* in particular is broadly structured into eastern, western, and central African genetic populations (Michel *et al*. 2005). In east and southern Africa, the Great Rift Valley has been hypothesized as a major barrier to gene flow, with genetically distinct *An. funestus* populations on either side of the valley (Kamau *et al*. 2002; Michel *et al*. 2005). Additionally, *An. funestus* populations across East and Southern Africa exhibit substantial genetic structuring. A broadly connected equatorial population spans from Ghana to Kenya, while populations south of the equator, including those in southeast Africa, are more genetically distinct from one another (Boddé *et al*. 2024). Across both northern and southern regions, multiple chromosomal inversion karyotypes are segregating and may play a role in local adaptation (Boddé *et al*. 2024). These complexities in gene flow patterns, population sub-structure, and ecological adaptations potentially impact the performance of current insecticidal interventions (Clarkson *et al*. 2018), or future biocontrol technologies such as gene-drive modified mosquitoes (Nolan 2021)

Recent discoveries have shown that mutations in the major metabolic resistance genes, *Cyp6p9a* and *Cyp6p9b,* are widespread in pyrethroid-resistant *An. funestus* mosquitoes from East and Southern Africa but absent in West and Central Africa (Mugenzi *et al*. 2019; Weedall *et al*. 2019). Conversely, resistance to DDT conferred by the glutathione S-transferase epsilon (*L119F-GSTe2*) mutation, is largely restricted to West and Central Africa (Tchigossou *et al*. 2020), with evidence of spread to Eastern Africa (Odero *et al*. 2024b), where localized DDT resistance is also conferred by *kdr* mutations in the *Vgsc* gene (Odero *et al*. 2024a). Our recent analysis of Tanzanian *An. funestus* revealed a south–north directional spread of the *Cyp6p9a* and *Cyp6p9b* genotypes; suggesting landscape feature-driven gene flow barriers or breakdown of past genetic barriers (Odero *et al*. 2024b), consistent with the recently observed selection at the *Cyp6* gene family (Boddé *et al*. 2024). However, despite this spatial genetic variation, phenotypic resistance profiles were broadly similar in Tanzanian *An. funestus* populations nationwide (Odero *et al*. 2024b).

In this current study, we analyzed the whole-genome sequences of 334 *An. funestus* mosquitoes sampled from 11 administrative regions with varying ecologies and malaria burden across mainland Tanzania. The aim was to understand how the vector populations are genetically structured, identify potential drivers of the genetic differences, and assess how genetic variation relates to observed insecticide resistance.

## Materials and methods

### Population sampling and sequencing

**Fig. 1.**
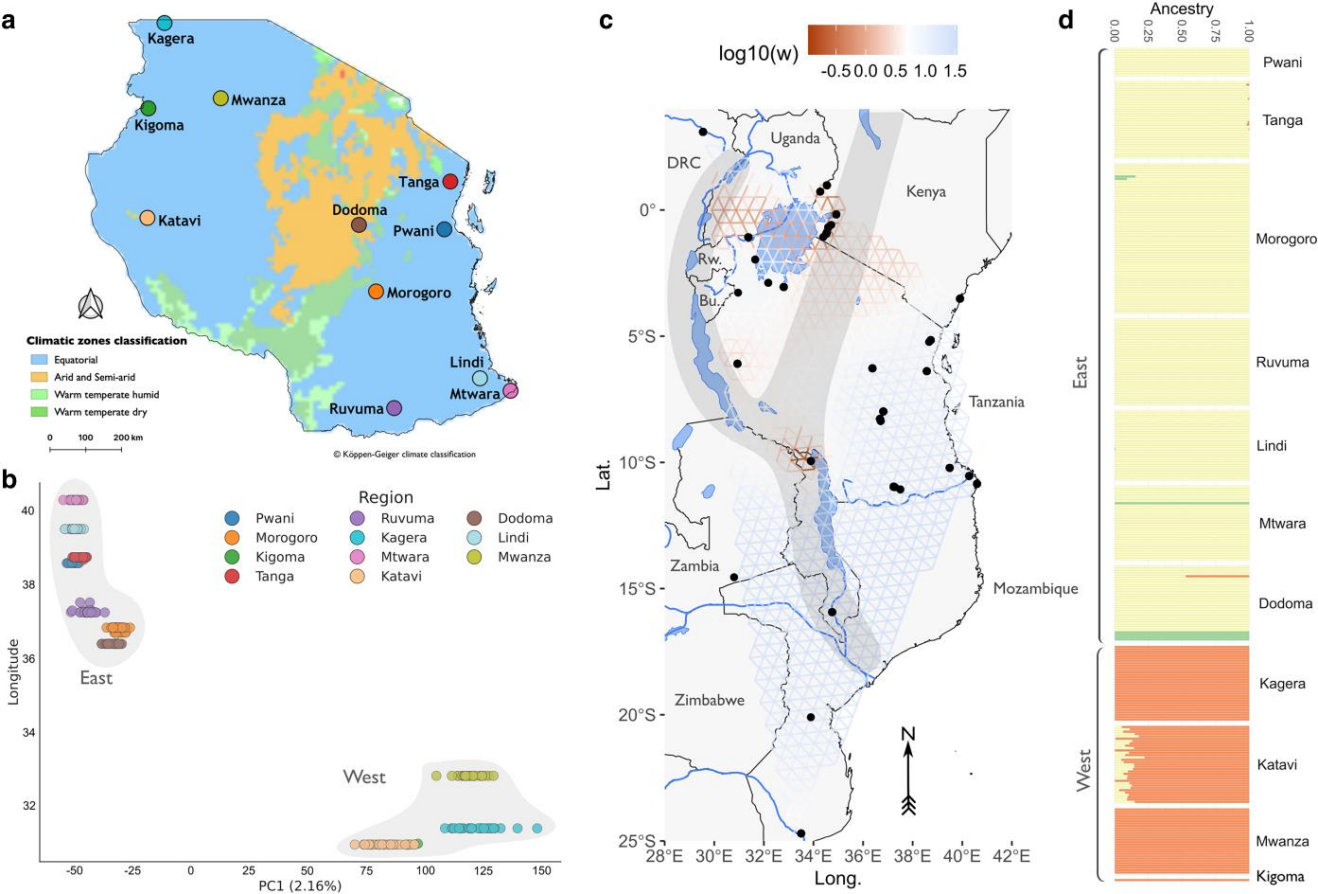
Population structure of *An. funestus* in Tanzania. a) is a map of Tanzania, with cohort sampling areas denoted by colored points. See Materials and methods and Supplementary Table 1 for more information on cohort sampling locations. Map is colored by Koppen–Geiger climate classification (see key). b) is a principal component analysis of all samples. The *x*-axis shows variation along and % of total variance explained by PC1, *y*-axis indicates longitude of sample collection. Points are colored by cohort (collection region). Shading indicates membership of East/West grouping. c) is the result of fEEMS, showing inferred migration between samples collected in Tanzania and the wider East African region (Boddé *et al*. 2024). Points denote individual sampling locations, the colored grid shoes inferred migration [log10(w)] of the best fitting model, where brown = low and blue = high. The approximate location of the Rift Valley system is shaded in grey. Countries are as labeled (DRC: Democratic Republic of Congo, Bu.: Burundi, Rw.: Rwanda). d) shows ADMIXTURE analysis of all samples, with the best fitting K (3). The *x*-axis indicates individual cluster membership, *y*-axis indicates individual sample. Plot is paneled by cohort, with membership of East or West indicated on the left-hand side.

The 344 female *An. funestus* specimens analyzed in this study were collected from 31 villages spanning the eco-geography of Tanzania as previously described in Odero *et al.* (2024a). Given the proximity of sampling locations within each region, we collapsed them into 11 analysis cohorts (regions) representing their geographic locations. (Fig. 1a & Supplementary Table 1). The collections formed part of a countrywide *An. funestus* surveillance project in Tanzania and were subsequently incorporated into the MalariaGEN *Anopheles funestus* genomic surveillance project database (https://www.malariagen.net/project/anopheles-funestus-genomic-surveillance-project/). Most mosquitoes were collected in households between 2021 and 2023 using CDC light traps and mechanical aspirators. They were sorted by sex and taxa and *An. funestus* group mosquitoes were preserved individually in 96-well plates containing 80% ethanol.

Genomic DNA was extracted from individual mosquitoes using DNeasy Blood and Tissue Kits (Qiagen, Germany). A single band confirmed the purity and integrity on 1% agarose gel and a minimum DNA concentration of 20 ng/μl on a Qubit® 2.0 fluorometer. Samples that passed quality control were individually commercially whole-genome-sequenced at 30×.

### Sequence analysis and single-nucleotide polymorphism calling

Detailed specifications of the sequence analysis, single-nucleotide polymorphism (SNP) calling, and haplotype phasing pipelines are available from the MalariaGEN GitHub repository (https://github.com/malariagen/pipelines/). Briefly, reads were mapped to the *An. funestus* reference genome idAnoFuneDA-416_04 (Ayala *et al*. 2022) with Burrows-Wheeler Aligner (BWA) version v0.7.15. Indel realignment was performed using Genome Analysis Toolkit (GATK) version 3.7-0 RealignerTargetCreator and IndelRealigner. SNPs were called using GATK version 3.7-0 UnifiedGenotyper. Genotypes were called for each sample independently, in genotyping mode, given all possible alleles at all genomic sites where the reference base was not “N”. The aligned sequences in BAM format were stored in the European Nucleotide Archive (ENA) under study number PRJEB2141.

High-quality SNPs and haplotypes were identified using BWA version 0.7.15 and GATK version 3.7-0. Quality control involved the removal of samples with low mean coverage, removing cross-contaminated samples, running PCA to identify and remove population outliers, and sex confirmation by calling the sex of all samples based on the modal coverage ratio between the X chromosome and the autosomal chromosome arm 3R. Full quality control methods are available on the MalariaGEN vector data user guide (https://malariagen.github.io/vector-data/ag3/methods.html). We used decision-tree filters that identify genomic sites where SNP calling and genotyping are likely to be less reliable. Genotypes at biallelic SNPs that passed the decision-tree site filtering process were phased into haplotypes using a combination of read-backed and statistical phasing. Read-backed phasing was performed for each sample using WhatsHap version 1.0 (https://whatshap.readthedocs.io/). Statistical phasing was then performed using SHAPEIT4 version 4.2.1 (https://odelaneau.github.io/shapeit4/).

### Population structure

To estimate geographic genetic population structure in *An. funestus* across Tanzania, we used principal component analysis (PCA) and a model-based ADMIXTURE ancestry analysis. PCA was conducted on Google Colab in the malariagen_data (v14.0.0) python API (https://malariagen.github.io/malariagen-data-python/latest/Af1.html), using the inversion-free region of chromosome 2RL: 57,604,655–90,000,000 obtained from the full data set via random down-sampling to 100,000 SNPs and plotted using the seaborn python package (Waskom 2021). ADMIXTURE analysis was performed to classify the individual mosquitoes of unknown ancestry into discrete populations. The analysis was performed using ADMIXTURE v1.3.0 (Alexander *et al*. 2009) on 100,000 SNPs randomly down-sampled from chromosome 2RL with *K* (a priori clusters) values ranging from 1 to 10. The best value of *K* with the lowest cross-validation error (CVE) of 0.10027 was *K* = 3 followed closely by *K* = 4 (CVE 0.10352) and *K* = 2 (CVE 0.10373) (Supplementary Fig. 1). ADMIXTURE was run using identical parameters across 5 different random seeds, and results were examined visually between runs to ensure consistency. The R package Starmie (https://github.com/sa-lee/starmie) was used to summarize the results and the output visualized using ggplot2 in R (Wickham 2009).

We estimated spatial population structure by inferring effective migration surfaces using Fast Estimation of Effective Migration Surfaces (fEEMS) models (Marcus *et al*. 2021). We used samples published in Boddé *et al*. (2024), from the wider East and Southern African region; Kenya, Uganda, Zambia, Mozambique, and the Democratic Republic of the Congo (DRC). The exact sample set IDs are specified in the notebook in the GitHub repository (https://github.com/joelodero/TZ_funestus_genomics_2025). 100,000 SNPs were randomly selected from the regions specified above and used as model inputs in fEEMS. Cross-validation was performed as per the package documentation, and the model with the lowest cross-validation error was selected. Final model outputs were plotted in R using the *rnaturalearth* (Massicotte and South 2025) package to plot physical features and country borders.

We further examined spatial population structure by estimating between-sample relatedness using PC-Relate (Conomos *et al*. 2016), implemented in sgkit (https://sgkit-dev.github.io/sgkit/latest/), and using a Mantel Test implemented in scikit-bio (Rideout *et al*. 2025) to test for correlation between inferred sample relatedness and geographic distance (https://geopy.readthedocs.io/en/stable/) matrices. Based on the outputs from the PCA, ADMIXTURE, and fEEMS, samples were grouped into populations (East or West, following PCA and ADMIXTURE clustering), or cohorts according to the sampling location and administrative region.

### Genetic diversity and population history

We computed nucleotide diversity (π) and Tajima's *D* on Google Colab following procedures outlined in the MalariaGEN Python package https://malariagen.github.io/malariagen-data-python/latest/Af1.html. We determined the signatures of recent inbreeding among individuals by estimating genome-wide runs of homozygosity (ROH). ROH were inferred using a hidden Markov model implemented in *scikit-allel* (Miles *et al*. 2024). For more information on the model, see the Supplementary Material of The Anopheles gambiae 1000 Genomes Consortium, 2017 (Anopheles gambiae 1000 Genomes Consortium *et al*. 2017). Due to the difficulty of interpreting short ROH (Pemberton *et al*. 2012), ROH < 100,000 bp in length were discarded.

We estimated changes in effective population size (*Ne*) over time by using *Stairwayplot2* (Liu and Fu 2020). We generated folded site-frequency spectra (SFS) for East and West populations by randomly sampling 5,000 SNPs each from chromosome 2RL: 57,604,655–90,000,000 and used them as input to *Stairwayplot2*. We ran *Stairwayplot2* using default parameters, assuming a generation time of 0.09 (11 per year) and mutation rate of 2.8E-9.

### Genome scans

Genome-wide *Fst* scans were used to determine the extent to which allele frequencies differ between groups of individuals and the degree of differentiation between the sampled populations. *Fst* was estimated per-chromosome using the *fst_gwss* function in the malariagen_data python API (https://malariagen.github.io/malariagen-data-python/latest/Af1.html) with a window size of 5,000 and a minimum cohort size of 10. We also searched for signatures of recent selection using Garud's H12 (Garud *et al*. 2015). H12 scans were performed on *An. funestus* genotypes by population where sample *n >* 10 and a window size of 5,000 using the *h12_gwss* function in the malariagen_data python API. For both, we validated the potential impact of unequal cohort representation (e.g. 64 samples from Morogoro) by performing the analyses with no more than 20 randomly selected samples from each cohort and found no difference (Supplementary Fig. 2).

### Diplotype clustering

To investigate selection and gene flow in *An. funestus,* we used diplotype clustering (Nagi *et al*. 2024) with all 334 samples at transcript LOC125764713_t1, 2RL: 8,685,464–8,690,407, and LOC125764232_t1, X: 8,339,269–8,341,975. The clustering pattern of diplotypes from an individual can be used to identify selective sweeps, and better captures CNVs, that can be challenging to phase. Diplotype clustering dendrograms for each transcript were plotted alongside per-sample heterozygosity over the locus, as well as CNV frequencies and non-synonymous mutations. For readability, we retained only SNPs segregating at frequencies > 0.01 over all populations.

### Resistance locus variation

To identify potential nucleotide polymorphisms in the *Cyp6p* and *Cyp9k1* genes, we extracted SNPs altering the amino acid of transcripts LOC125764713_t1 and LOC125764232_t1. We computed allele frequencies on mosquito cohorts defined by the region and collection year. We plotted mutations segregating at frequencies >0.05 in one or more cohorts (Anopheles gambiae 1000 Genomes Consortium *et al*. 2017). CNV frequencies were computed for the *Cyp6p* gene cluster (*Rp1*) (2RL: 8.6–8.8 Mbp) and *Cyp9k1* genes (X: 8.35–8.55 Mbp) using the *gene_cnv_frequencies* function in the malariagen_data python API. Full details of CNV calling and phasing are available on the MalariaGEN Python package (https://malariagen.github.io/malariagen-data-python/latest/Af1.html).

## Results

### Population structure

PCA of chromosome 2RL revealed two main clusters (Fig. 1b & Supplementary Fig. 3a). The first cluster contained *An. funestus* cohorts from western, higher altitude regions of Katavi, Kigoma, Kagera, and Mwanza (hereafter referred to as the “West” population). The second cluster contained coastal cohorts from eastern lower altitude areas of Pwani, Morogoro, Tanga, Ruvuma, Mtwara, Dodoma, and Lindi (hereafter, “East”) (Fig. 1b). The PCA based on chromosome X similarly showed the West and East genetic clusters, but the samples from West were dispersed along PC1 and PC2 (Supplementary Fig. 3b). In population-specific PCA, samples from West Tanzania remained in a single cluster but with some dispersed samples from the Kagera region (Supplementary Fig. 3c). However, the second cluster containing East samples was separated into three genetic clusters with a clear pattern of geographic separation (Supplementary Fig. 3d).

We used fEEMS to investigate the spatial genetic structure of *An. funestus* across the East African region (see Materials and methods). Overall, samples from the southeast of the range (including East cohorts, Zambia, Mozambique, southern Kenya and Malawi) appeared well-connected with respect to each other (Fig. 1c). These were separated by a region of reduced migration from other samples from West, the DRC, northern Malawi, and western Kenya and Uganda. These cohorts showed signs of relatively low migration between them (Fig. 1c). The Tanzania-wide sample set showed a significant decay in genetic relatedness over distance between samples (Mantel coefficient: −0.04, *P* value: 0.001) (Supplementary Fig. 4). The ADMIXTURE analysis further supported separation between East and West Tanzania, with the best fitting value of *K* = 3 supporting two major groups, corresponding to West and East (Fig. 1d).

### Genome-wide population diversity

**Fig. 2.**
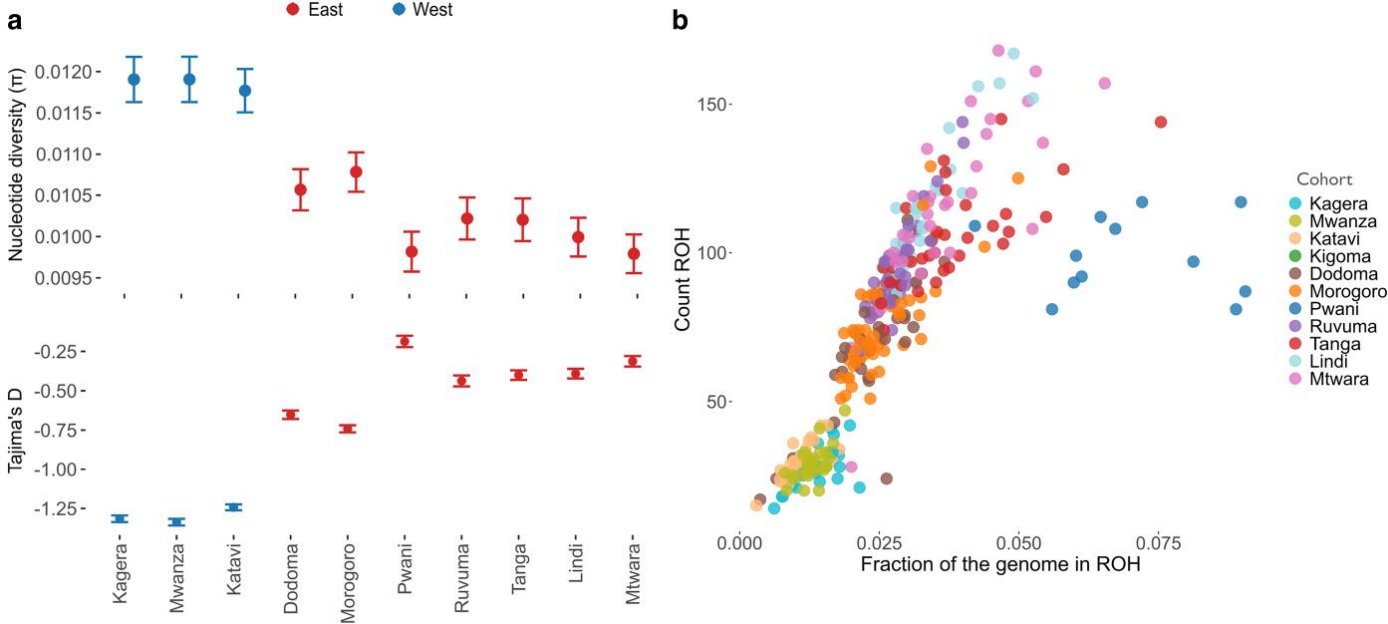
Genetic diversity in *An. funestus* populations in Tanzania. a) Nucleotide diversity (*π*) and Tajima's *D* calculated using SNPs from chromosome 2RL. Points and whiskers (denoting 95% CI) are calculated by cohort and colored by membership of East or West. b) Scatterplot showing the number of ROH (*y*-axis) and fraction of the genome in ROH (*x*-axis) in individual mosquitoes. Each marker represents a mosquito and is colored by cohort (region).

To determine genome-wide population diversity, we computed nucleotide diversity (π) and Tajima's *D* (Tajima 1989) (see Materials and methods). Nucleotide diversity (π) was significantly higher in the West samples compared to the East populations (*t*-statistic = −11.27, *P* < 0.0005) (Fig. 2a). Similarly, Tajima's *D* was significantly lower in the West samples compared to the East populations (*t*-statistic = 10.96, *P* < 0.0005) (Fig. 2a). We further investigated population history by examining ROH and effective population size (*Ne*). The proportion of the individual genome in long ROH is informative of recent demographic processes (Ceballos *et al*. 2018). ROH are stretches of the genome where identical haplotypes are inherited from each parent due to inbreeding or recent common ancestry (Ceballos *et al*. 2018). Individuals from the West population (Kagera, Katavi, Kigoma, and Mwanza) generally had fewer ROH segments and a lower fraction of their genomes in ROH than from East (Dodoma, Morogoro, Pwani, Ruvuma, Tanga, Lindi, and Mtwara) which generally had more ROH segments and a higher fraction of their genomes in ROH (Fig. 2c & Supplementary Fig. 5a). Despite these differences in diversity and inbreeding, *Stairwayplot2* analysis showed that eastern and western cohorts had similar population histories, with a gradual decline in effective population size (*Ne*) since an apparent peak approx. 1 mya (Fig. S5b), suggesting that these differences in diversity and inbreeding may reflect more recent population trends, rather than long term differences in population history.

### Genome-wide signatures of selection

**Fig. 3.**
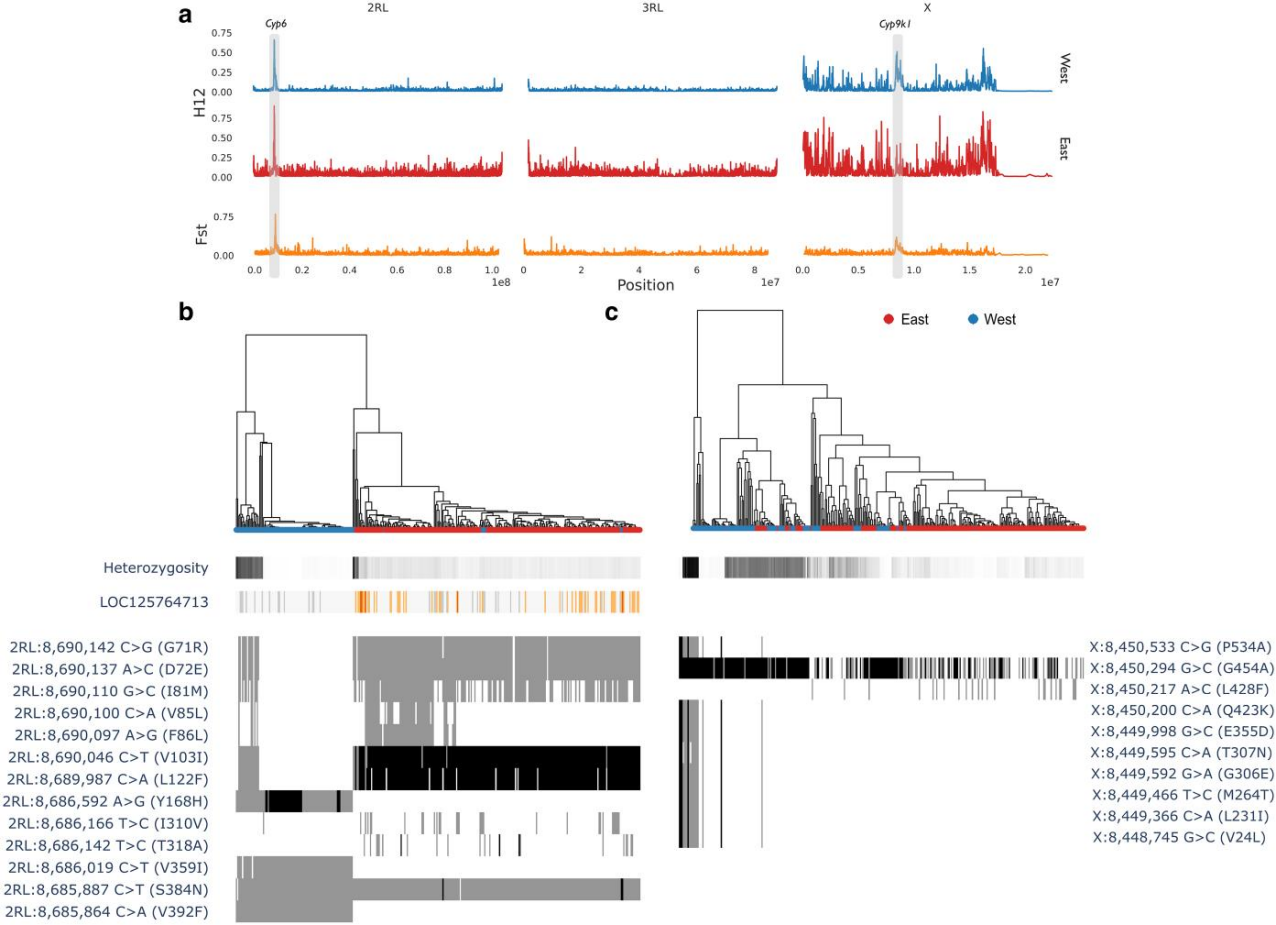
Genome-wide signatures of selection. a) Windowed haplotype homozygosity (H12) genome-wide selection scans at along chromosomes 2RL, 3RL, and X. The *x*-axis indicates the position [in base pairs (bp)], and the *y*-axis indicates the H12. The H12 values range between 0 and 1, where zero indicates high haplotype diversity within a genomic window, and values closer to one indicate a strong selection of dominant haplotypes. Plots are paneled by East and West. Windowed Fst between East and West along chromosomes 2RL, 3RL, and X. The *x*-axis indicates the position [in base pairs (bp)], and the *y*-axis indicates the fixation index (Fst). Haplotype clustering over the *Cyp6p9a* (b) and Cyp9k1 (c) genes. Dendrogram length indicates cityblock distance clusters with zero genetic distances indicate a selective sweep. Leaf color denotes individual membership of East or West. Bars below indicate locus heterozygosity and copy number of *Cyp6p9* (no CNVs were detected over *Cyp9k1*). The bar plots indicate the presence, and zygosity (grey: heterozygous, black: homozygous) of non-synonymous mutations segregating at > 5% in all samples. Labels indicate chromosome, position, and amino acid change.

We searched for regions of the genome under possible recent selection with genome selection scans (GWSS) of the H12 statistic (Garud *et al*. 2015). Following the identification of genetic clustering (as shown in Fig. 1b-d), the analysis populations for GWSS were categorized into West (found in samples from Katavi, Kigoma, Kagera, and Mwanza) and East (found in samples from (Tanga, Morogoro, Pwani, Dodoma, Ruvuma, Lindi, and Mtwara). GWSS of H12 revealed a clear signal of elevated H12 at the *Cyp6* gene cluster in both populations (Fig. 3a). *Cyp9k1* had a clear H12 elevation in the West genetic cluster, but possible peaks of elevated H12 near *Cyp9k1* in East were unclear against the relatively higher genome-wide H12 background (Fig. 3a). To assess the extent to which these selective sweeps may be shared between populations, and the level of genome-wide differentiation more generally, we used a GWSS of *Fst* between East and West (Fig. 3a). *Fst* values genome-wide were generally low (0.035) with high peaks (*Fst* ∼0.8) between West and East around the genomic regions containing the *Cyp6* gene cluster on chromosome 2RL, and *Cyp9k1* on chromosome X, indicating strong differentiation at these regions, and that the selective sweeps at these loci in East and West are driven by different alleles Fig. 3a). We explored the evolution of these regions further by plotting diplotype clustering dendrograms, locus heterozygosity, CNV and non-synonymous mutation frequencies for the genes *Cyp6p9* (Fig. 3b) and *Cyp9k1* (Fig. 3c). For readability, we only plotted mutations segregating at a frequency of >5% over the whole population. The dendrogram for the *Cyp6p9* gene revealed two major clades (Fig. 3b). The first clade contained several identical diplotypes from the western cohorts, the second contained mostly identical diplotypes from the eastern cohorts. Distinct CNV profiles and sets of non-synonymous mutations were present for each clade (Fig. 3b), with elevated copy number particularly pronounced in East, likely accounting for the high number of apparently non-synonymous mutations segregating at this gene. Mutations V103I and L122F were homozygous and almost fixed in East, alongside heterozygous G71R, D72E, I81M and S384N. By contrast, V103I and L122F were absent from east. Instead, heterozygous Y168H, V359I, S384N and V392F dominated (Fig. 3b). The diplotype clustering dendrogram for *Cyp9k1* showed a similar, if less-pronounced, separation between diplotypes from East and West samples. We observed higher frequencies of all non-synonymous mutations including, notably, the G454A mutation (Tagne *et al*. 2025), in West and no evidence for copy number variation at this locus (Fig. 3c).

### Resistance locus variation

Evidence of distinct resistance architectures in East and West was further supported by amino acid variation and CNV frequencies across the *Cyp6* cluster. We searched for non-synonymous amino acid variation in the *Cyp6* cluster and *Cyp9k1,* filtering on 5% minimum allele frequencies in any cohort. We discovered 117 non-synonymous variants across the two (Supplementary Table 2). We further analyzed transcript LOC125764713_t1, containing *Cyp6p9a* which is crucial for insecticide detoxification in *An. funestus* populations from east and southern Africa (Mugenzi *et al*. 2019; Weedall *et al*. 2019). The *Cyp6p9a* gene had 13 non-synonymous amino acid variations, that followed the East:West separation showed by the diplotype clustering (Supplementary Fig. 6a) with notably high frequencies of V103I (86–100%) and L122F (87–100%), both almost fixed in the East cohorts, (Fig. 3b, Supplementary Fig. 6a) but absent in two West cohorts (Kagera and Mwanza) and only at 32% frequency in Katavi (Supplementary Fig. 6a). Three additional variants, Y168H, V392F, and V359I, were present in the West cohorts at > 40% frequency but were absent in the East cohorts (Supplementary Fig. 6a). Further variants were detected in the *Cyp6p9a* gene in cohorts nationwide without a discernible geographical pattern (Supplementary Fig. 6a).

The *Cyp9k1* gene, transcript LOC125764232_t1, had 11 amino acid variants, with variable frequencies of G454A across all populations (Supplementary Fig. 6b). G454A was at near fixation (98%) in samples collected from the West cohort (Katavi, Kagera, and Mwanza) (Supplementary Fig. 6b). Two East populations, Dodoma and Morogoro (collection year 2021), also had at least 80% frequency of G454A, with the other East populations (Tanga, Ruvuma, Mtwara and Lindi) having between 10 and 36% (Supplementary Fig. 6b). In Morogoro, the frequency of the G454A mutation showed an increasing trend over 4 years, peaking at 87% in 2023, though this trend was insignificant (*P* = 0.06627, Supplementary Fig. 6b).

We further investigated CNV frequencies of amplifications (increase in copy number) and deletions (reduction in copy number) at the *Cyp6p* gene cluster (*Rp1*) and *Cyp9k1* genes. In the *Cyp6* cluster, CNVs around the probable cytochrome P450 6a13 gene (transcript LOC125764726) had the highest amplification frequency of >87% in all Tanzanian cohorts (Supplementary Fig. 7a). The esterase E4-like gene (transcript LOC125764700) amplification ranged from 30 to 70% with moderate CNV deletions in the West cohorts (Supplementary Fig. 7a). CNVs on the *Cyp6p9a* (transcript LOC125764713) were found in three East cohorts (Mtwara, Lindi and Ruvuma) (Supplementary Fig. 6a). However, we did not find evidence of CNV amplification/deletion on the *Cyp9k1* gene in any populations (Supplementary Fig. 7b).

## Discussion

Our findings demonstrate the existence of two genetically differentiated *An. funestus* populations in Tanzania; one comprising cohorts from the east of the country, and the other from the west. These populations show differences in diversity and inbreeding, as well as signs of isolation by distance and continuous spatial structure corresponding to the arid, elevated centre of the country, occupied by the Rift Valley. Interestingly, despite low genome-wide differentiation overall we found evidence of asynchronous selective sweeps at distinct insecticide resistance gene alleles in east and west, likely driven by different CNV alleles. This corroborates recent resistance genotyping data (Odero *et al*. 2024b) and suggests that different resistance architectures underly a consistent resistance phenotype across the country.

It seems likely that East and West are part of the broader equatorial and southeast African *An. funestus* super-populations (Boddé *et al*. 2024). The Rift Valley, which lies between them, has long been postulated as a gene flow barrier across the east and southern African region in *An. gambiae* (Lehmann *et al*. 1999; Michel *et al*. 2005), and *An. funestus* in Malawi (Barnes *et al*. 2017) and Kenya (Ogola *et al*. 2019). Tanzania's climate is largely equatorial, with an arid desert cutting across the central part of the country (Beck-Johnson *et al*. 2013). These harsh arid conditions in central Tanzania are potentially less favorable to *An. funestus* and may thus also influence their genetic structure. Despite separation on PCA, differences in diversity and selection between East and West, low genome-wide *Fst,* and fEEMS results indicating some, if reduced, migration in this region suggest that this is not an impermeable barrier. The apparent paucity of *An. funestus* from this part of the country (*An. arabiensis* is the dominant vector species instead) (Mwalimu *et al*. 2024), both support it as a region serving as a barrier to gene flow, while making it challenging to disentangle the specific ecological or landscape features. A trend of increasing aridity in East Africa overall (Owen *et al*. 2018), may also explain signs of gradual *Ne* decline over the last million years in *An. funestus*. Comparable patterns of geographical and ecological isolation influencing gene flow in *An. funestus* have been described in Cameroon and Burkina Faso, where population structure correlates with chromosomal inversion polymorphisms and ecological zones such as savannah vs forest habitats (Michel *et al*. 2005; Ayala *et al*. 2011), and the potential impact of such geographic and climatic stratifications on local vector adaptation and genetic differentiation in Tanzania, and their implications for malaria transmission (Smith *et al*. 2024) should be investigated further, with greater sampling effort focussed on recovering *An. funestus* from this central region.

The strongest differentiation between the two populations was around the metabolic resistance genes (*Cyp9k1* and the *Cyp6p* cluster); indicating these genes are undergoing independent selective sweeps. For instance, analysis of the *Cyp6p9* gene demonstrated that V103I and L122F mutations were widespread and at high frequencies in the East *An. funestus* populations while the West populations had Y168H, V392F, and V359I. However, these mutations have less impact on insecticide resistance phenotypes in this vector across Tanzania (Odero *et al*. 2024b). The selective sweeps in the *Cyp6p9* gene coincided with the patterns of distribution of key metabolic resistance alleles in Tanzania with *Cyp6p9a* and *Cyp6p9b* observed as fixed in the East populations of the south and east but either at low frequencies or undetectable in the West populations of north and west Tanzania (Odero *et al*. 2024b). The structured allelic variation around genomic regions containing crucial metabolic resistance genes in *An. funestus* suggests underlying differences in the molecular basis of insecticide resistance across Tanzania. Notably, similar patterns have been reported in Central African *An. funestus* populations, where distinct resistance haplotypes (e.g. around *Cyp6p9a/b* and *Cyp9k1*) have become fixed or nearly fixed in some areas (Mugenzi *et al*. 2019; Weedall *et al*. 2019; Tagne *et al*. 2025). These findings support a continent-wide trend of parallel local adaptation under insecticide pressure, driven by strong selective sweeps that are often region or taxon specific.

The cytochrome P450 G454A-*Cyp9k1* mutation was near fixation in the West populations but present only at low frequencies in the East populations. This mutation has been demonstrated to confer phenotypic resistance to type I (permethrin) and II (deltamethrin) pyrethroids, commonly used on ITNs (Djoko Hearn *et al*. 2022; Tagne *et al*. 2025). Continentally, G454A-*Cyp9k1* is widespread in central Africa (Cameroon) and East Africa including Uganda (Djoko Tagne *et al*. 2025). We speculate that the presence of G454A-*Cyp9k1* at near fixation in West Tanzania may thus be part of a wider Central-East African resistance cline, suggesting historical connectivity between western Tanzania and the Central African corridor. Conversely, the East populations could be connected more to the southern African region where this mutation is either absent or at low frequencies (Tagne *et al*. 2025).

Our findings provide useful information when considering the implementation of transgenic vector control strategies such as gene drives and sterile insect releases in Tanzania. The divergence between the West and East *An. funestus* populations with limited gene flow between them indicate that multiple strategic release sites across the potential genetic barriers may be required for nationwide implementation.

## Conclusion

Overall, this study provides a comprehensive population genomic description of *An. funestus* in Tanzania. We demonstrate that the vector is differentiated into two genetically distinct populations with historical and contemporary gene flow patterns. We also reveal directional sweeps of haplotypes, especially in the *Cyp6* gene cluster, and geographic allelic variation in the genomic regions around the insecticide resistance genes *Cyp6p* and *Cyp9k1*. The genomic dataset generated in this study, accessible through the European Nucleotide Archive under study number PRJEB2141, will be crucial to expanding capabilities for molecular surveillance of insecticide resistance in *An. funestus* Tanzania.

## Supplementary Material

iyaf117_Supplementary_Data

## Data Availability

The scripts and Jupyter Notebook used to analyze genotype and haplotype data, and produce figures and tables are available from the GitHub repository: https://github.com/joelodero/TZ_funestus_genomics_2025. The raw read data generated in this study have been deposited in the European Nucleotide Archive (https://www.ebi.ac.uk/ena/browser/home) under study number PRJEB2141. Mature SNP, haplotype, and CNV data are available in the MalariaGEN Vector Observatory malariagen_data API (sample sets detailed in the GitHub repository). Supplemental material available at GENETICS online.
