# Supplementary material for: Genomic evidence of spatially structured gene flow and divergent insecticide resistance backgrounds of the malaria vector Anopheles funestus in Tanzania
Source: Genetics. 2025 Jul 7;230(4):iyaf117. doi: 10.1093/genetics/iyaf117 (PMC12341887; doi:10.1093/genetics/iyaf117)
Supplement: iyaf117_Supplementary_Data [file iyaf117_supplementary_data.zip › Supplementary_Figure_and_Table_Legends_GENETICS-2025-308116.docx]

**Supplementary Figures**

**Figure S1.** Validation of the best ADMIXTURE value of *K* with the lowest cross-validation error (CVE).

**Figure S2.** Genome-wide signatures of selection for East and West populations. Cohorts have been randomly down sampled to a maximum of 20 samples. (a) Windowed haplotype homozygosity (H12) genome-wide selection scans at along chromosomes 2RL, 3RL, and X. X-axis indicates the position (in base pairs (bp)), and the Y-axis indicates the H12. The H12 values range between 0 and 1, where zero indicates high haplotype diversity within a genomic window, and values closer to one indicate a strong selection of dominant haplotypes. Plots are panelled by East and West. (b) Windowed Fst between East and West along chromosomes 2RL, 3RL, and X. The X-axis indicates the position (in base pairs (bp)), and the Y-axis indicates the fixation index (Fst).

**Figure S3.** The population structure of *An. funestus* in Tanzania. (a) PCA plot of the first two principal components from the 2RL chromosome (b) PCA plot of the first two principal components from the X chromosome. (c) plot of the first two principal components from the 2RL chromosome for samples from west of the Rift Valley (d) plot of the first two principal components from the 2RL chromosome for samples from east of the Rift Valley. The colours denote the sampling cohorts (regions).

**Figure S4.** Isolation by distance (IBD) plot. Genetic relatedness over distance between all 334 samples analysed in this study.

**Figure S5.** a) Box plot showing the average number of runs of homozygosity per population across Tanzania. b) Stairway plot of population histories of eastern and western *An. funestus* populations.

**Figure S6.** Amino acid variation around the *Cyp6p9* and *Cyp9k1* genes. (a) Heatmap of *Cyp6p9a* gene allele frequencies. (b) Heatmap of *Cyp9k1* gene allele frequencies. On both plots, the Y-axis labels indicate mutation effect, chromosome position, and nucleotide change. X-axis labels indicate the collection date and heatmap intensity indicates frequency where darker = higher, with frequency labelled in each heatmap facet. Plot is panelled by cohort, with membership of East or West indicated at the bottom.

**Figure S7.** Copy number variations (CNV) in the major resistance gene family, *Cyp6p* (a) *and Cyp9k1* (b), across Tanzania. The Y-axis labels indicate the CNV with amplification indicated by ‘amp’ and deletion by ‘del’. X-axis labels indicate the collection date and heatmap intensity indicates frequency where darker = higher, with frequency labelled in each heatmap facet. Plot is panelled by cohort, with membership of East or West indicated at the bottom.

**Supplementary Tables**

**Table S1.** List of samples analysed in this study.

**Table S2.** Non-synonymous amino acid variation in the *Cyp6* cluster and *Cyp9k1* gene.
