# Supplementary figures and images for: Genomic evidence of spatially structured gene flow and divergent insecticide resistance backgrounds of the malaria vector Anopheles funestus in Tanzania
Source: Genetics. 2025 Jul 7;230(4):iyaf117. doi: 10.1093/genetics/iyaf117 (PMC12341887; doi:10.1093/genetics/iyaf117)

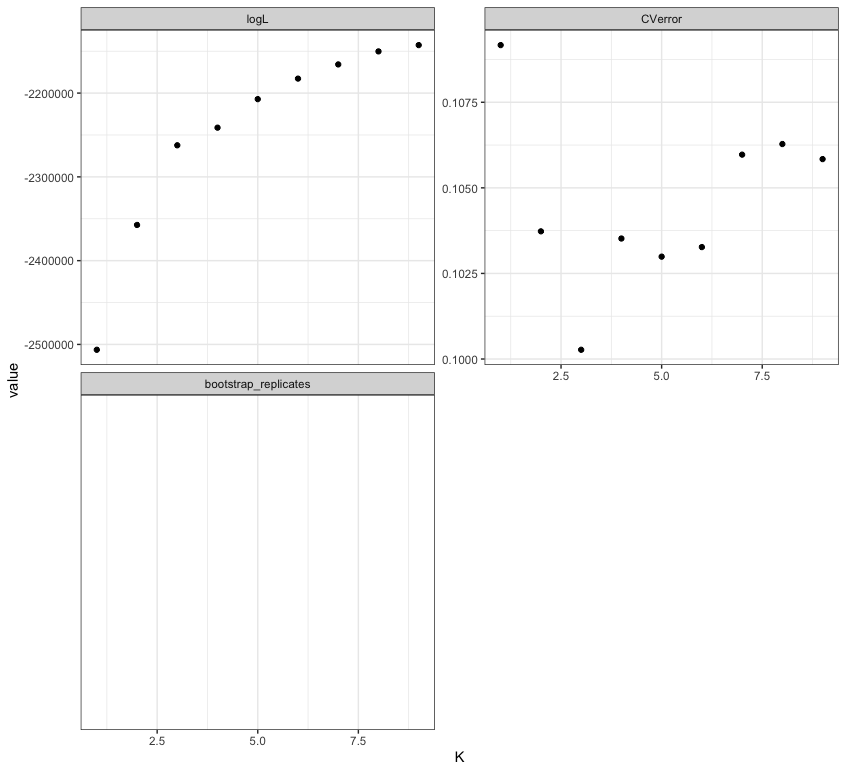

Supplement: iyaf117_Supplementary_Data [file iyaf117_supplementary_data.zip › Figure_S1_GENETICS-2025-308116.png]

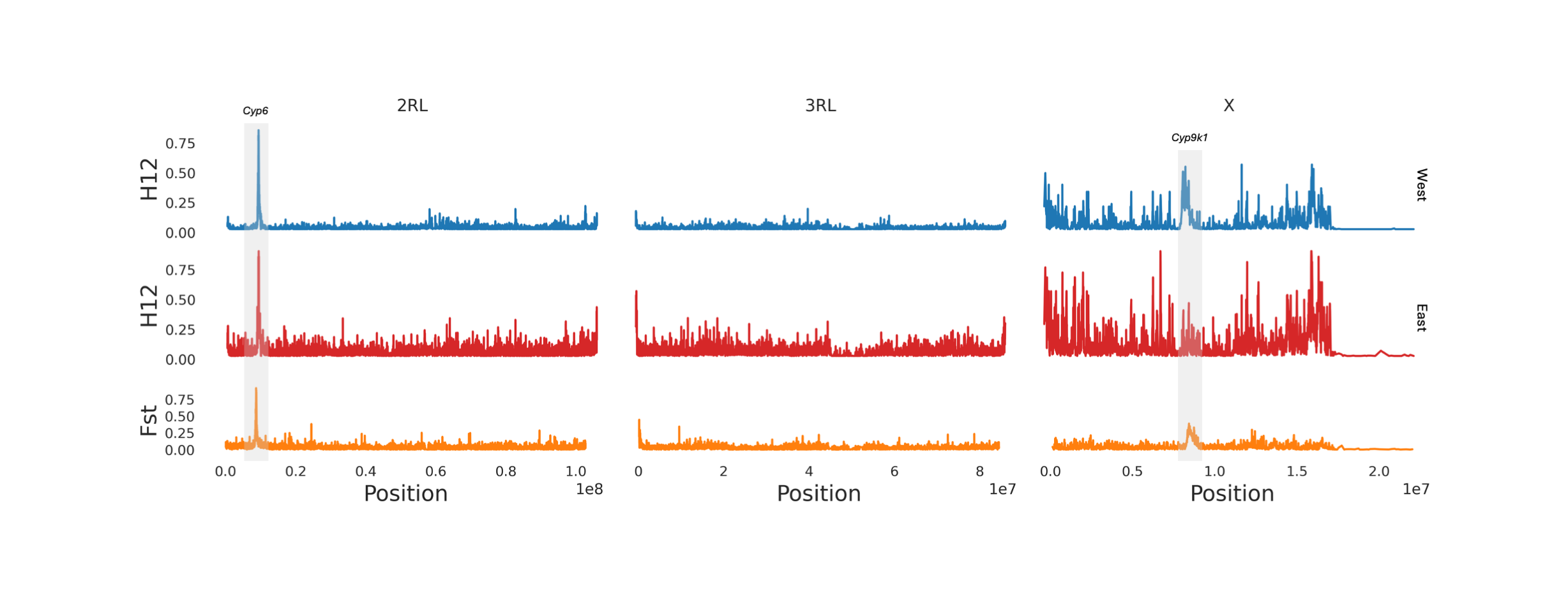

Supplement: iyaf117_Supplementary_Data [file iyaf117_supplementary_data.zip › Figure_S2_GENETICS-2025-308116.png]

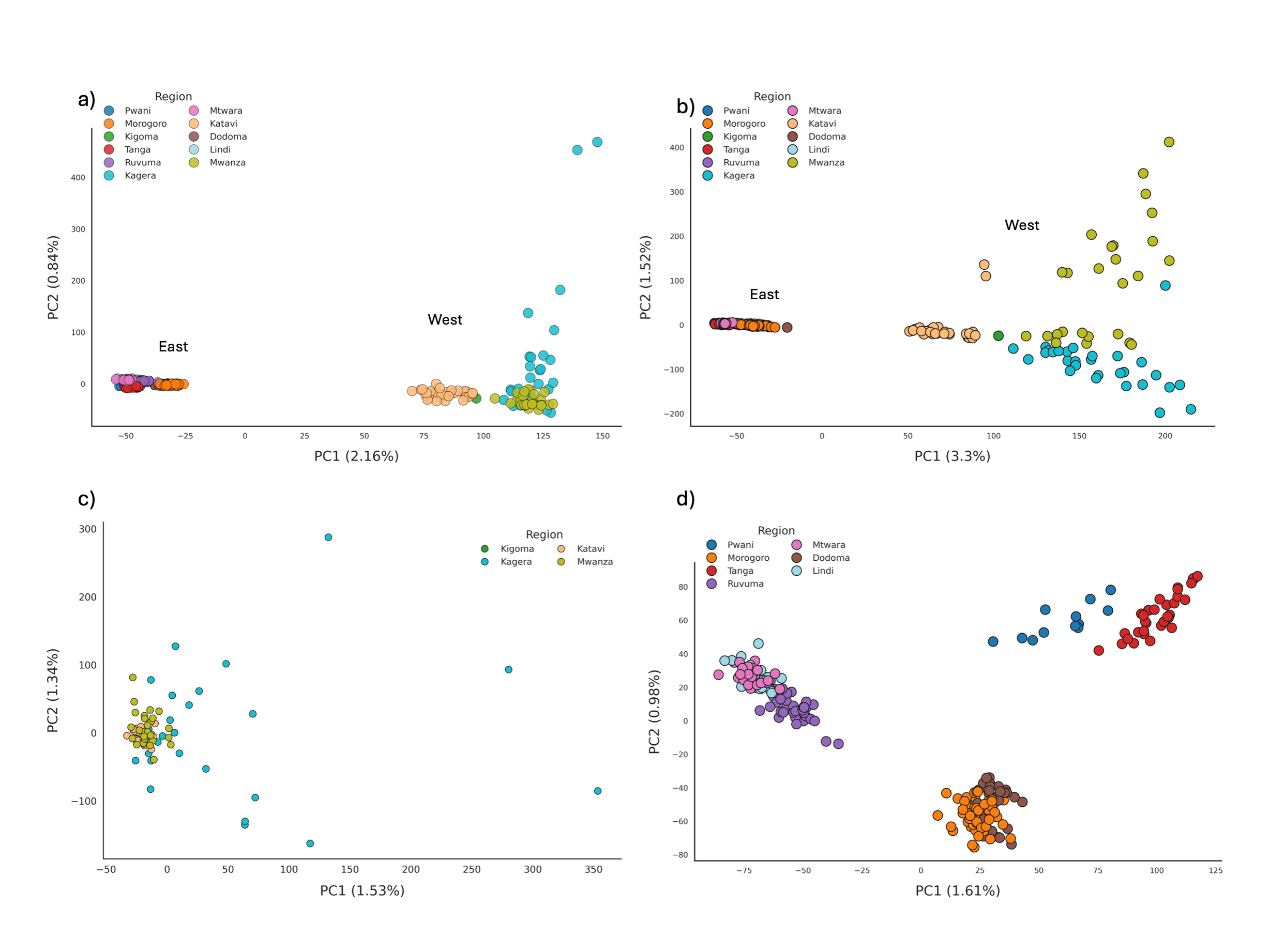

Supplement: iyaf117_Supplementary_Data [file iyaf117_supplementary_data.zip › Figure_S3_GENETICS-2025-308116.png]

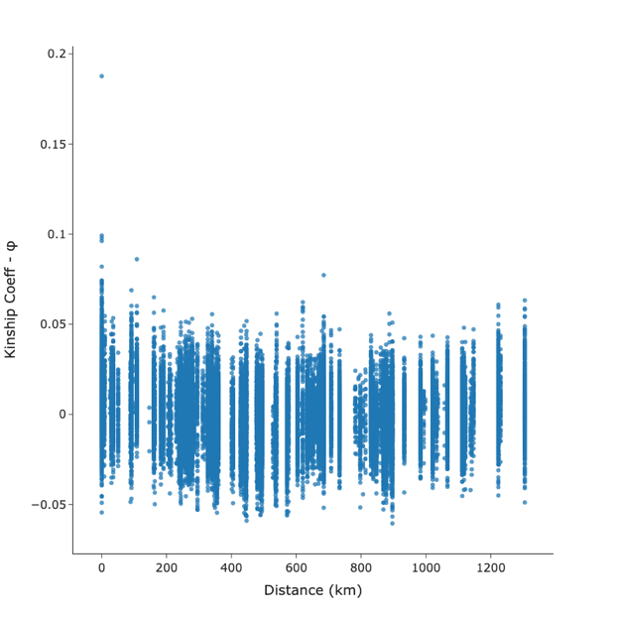

Supplement: iyaf117_Supplementary_Data [file iyaf117_supplementary_data.zip › Figure_S4_GENETICS-2025-308116.png]

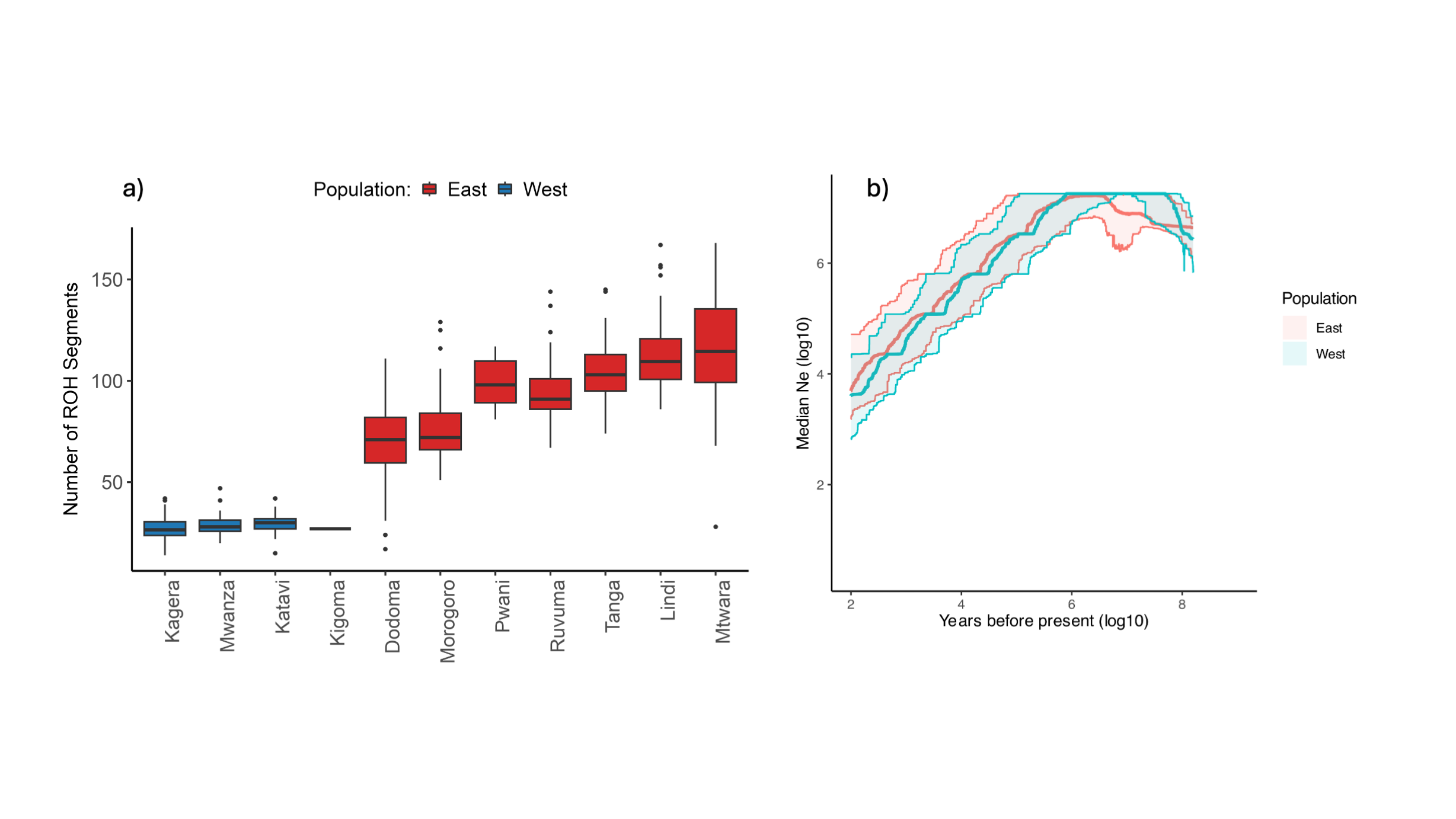

Supplement: iyaf117_Supplementary_Data [file iyaf117_supplementary_data.zip › Figure_S5_GENETICS-2025-308116.png]

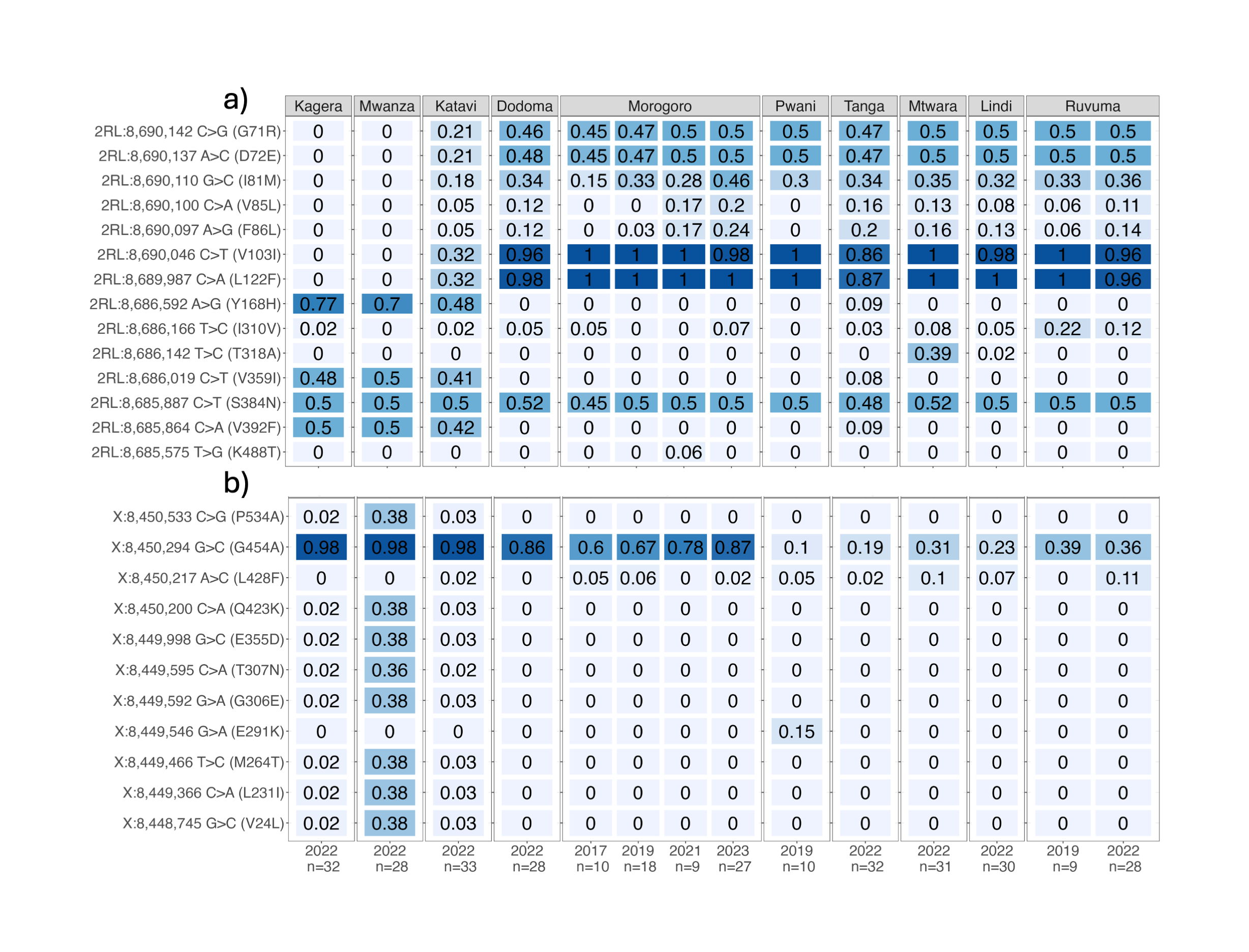

Supplement: iyaf117_Supplementary_Data [file iyaf117_supplementary_data.zip › Figure_S6_GENETICS-2025-308116.png]

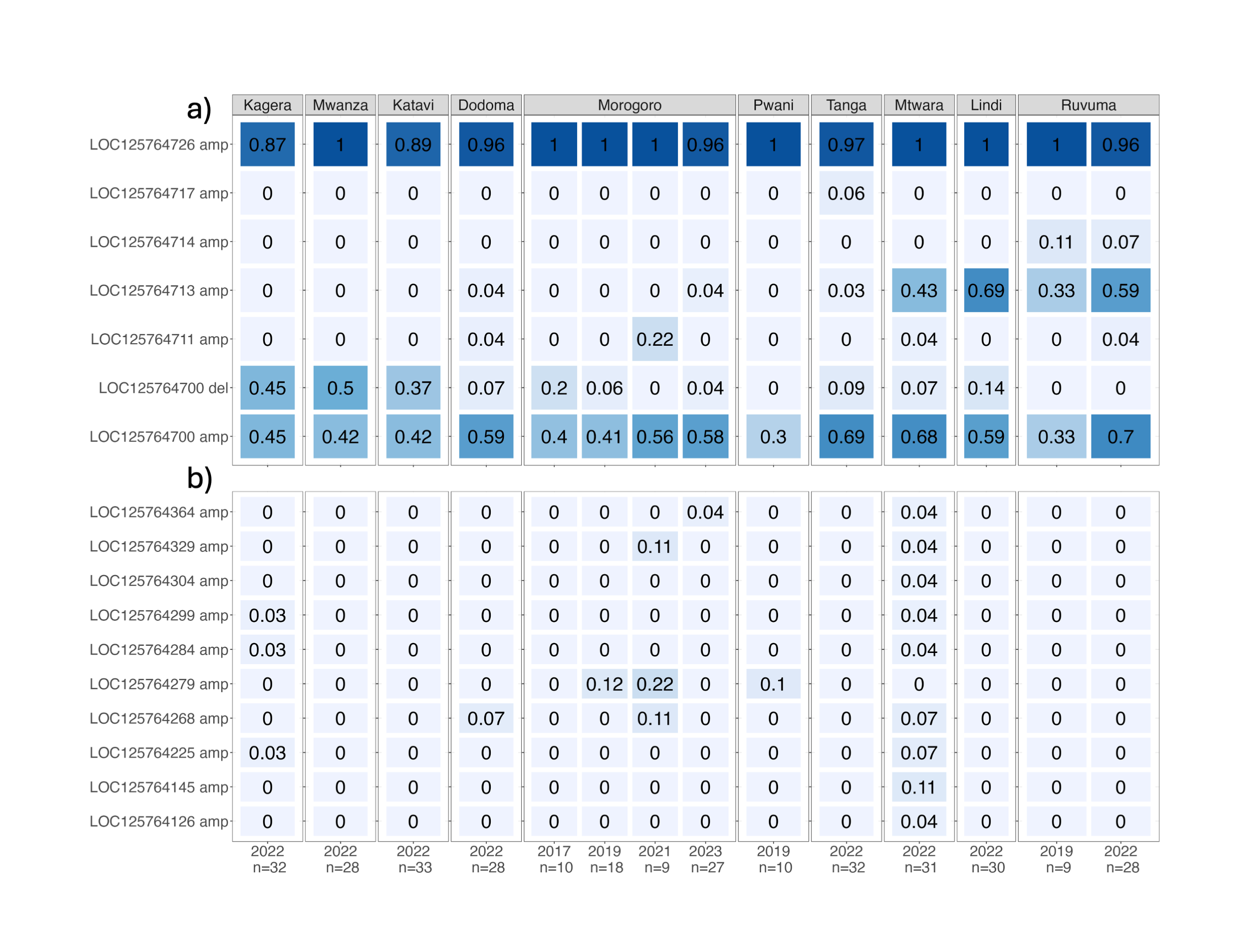

Supplement: iyaf117_Supplementary_Data [file iyaf117_supplementary_data.zip › Figure_S7_GENETICS-2025-308116.png]
